# *In situ* monitoring of hydrothermal reactions by X-ray diffraction with Bragg–Brentano geometry

**DOI:** 10.1107/S1600576720006019

**Published:** 2020-06-18

**Authors:** Karsten Mesecke, Winfried Malorny, Laurence N. Warr

**Affiliations:** a Hochschule Wismar, Philipp-Müller-Strasse 14, 23966 Wismar, Germany; b University of Greifswald, Friedrich-Ludwig-Jahn-Strasse 17A, 17489 Greifswald, Germany

**Keywords:** *in situ* X-ray diffraction, Bragg–Brentano geometry, hydrothermal reactions, tobermorite, autoclaved aerated concrete

## Abstract

This note describes an autoclave chamber developed and constructed by Anton Paar and its application for *in situ* experiments under hydrothermal conditions. Reactions of crystalline phases can be studied by successive *in situ* measurements on a conventional laboratory X-ray diffractometer with Bragg–Brentano geometry at temperatures <483 K and saturated vapour pressure <2 MPa. Variations in the intensity of X-ray diffraction reflections of both reactants and products provide quantitative information for studying the reaction kinetics of both dissolution and crystal growth. Feasibility is demonstrated by studying a cementitious mixture used for autoclaved aerated concrete production. During a period of 5.7 h at 466 K and 1.35 MPa, the crystallization of torbermorite and the partial consumption of quartz were monitored.

## Abstract 2

A feasibility study is carried out of an autoclave chamber for *in situ* X-ray diffraction experiments under hydrothermal conditions. Quartz dissolution and tobermorite formation are monitored on conventional laboratory X-ray diffractometers.

## 1. Introduction   

A wide range of commercial and non-commercial reaction cells have been developed for studying chemical reactions *in situ* by X-ray or neutron diffraction (Van Beek & Pattison, 2019 ▸), but so far hydrothermal reactions have been studied using such techniques exclusively in *transmission* geometry (Norby, 2006 ▸). Such transmission cells can be obtained by modifications of laboratory autoclaves (Van Beek & Pattison, 2019 ▸) and are suited to studying the formation of tobermorite and other calcium silicate hydrates at saturated vapour temperatures of 463 K (Bernstein, 2011 ▸), 463–583 K (Shaw *et al.*, 2000 ▸) and 463 K (Kikuma *et al.*, 2010 ▸; Matsui *et al.*, 2011 ▸). However, beamline time is limited and the lengths of these experiments vary between 5 h (Shaw *et al.*, 2000 ▸) and 13.5 h (Matsui *et al.*, 2011 ▸). Cells used to study these hydrothermal reactions with conventional laboratory X-ray diffractometers and *reflection* geometry have not yet been documented. This laboratory note details such a cell, which was developed by Anton Paar (Anton Paar GmbH, Graz, Austria), and the accompanying analytical setup used for *in situ* monitoring of tobermorite formation in association with the production of autoclaved aerated concrete (AAC).

## 2. Experimentation   

### 2.1. The device   

**Figure 1:**
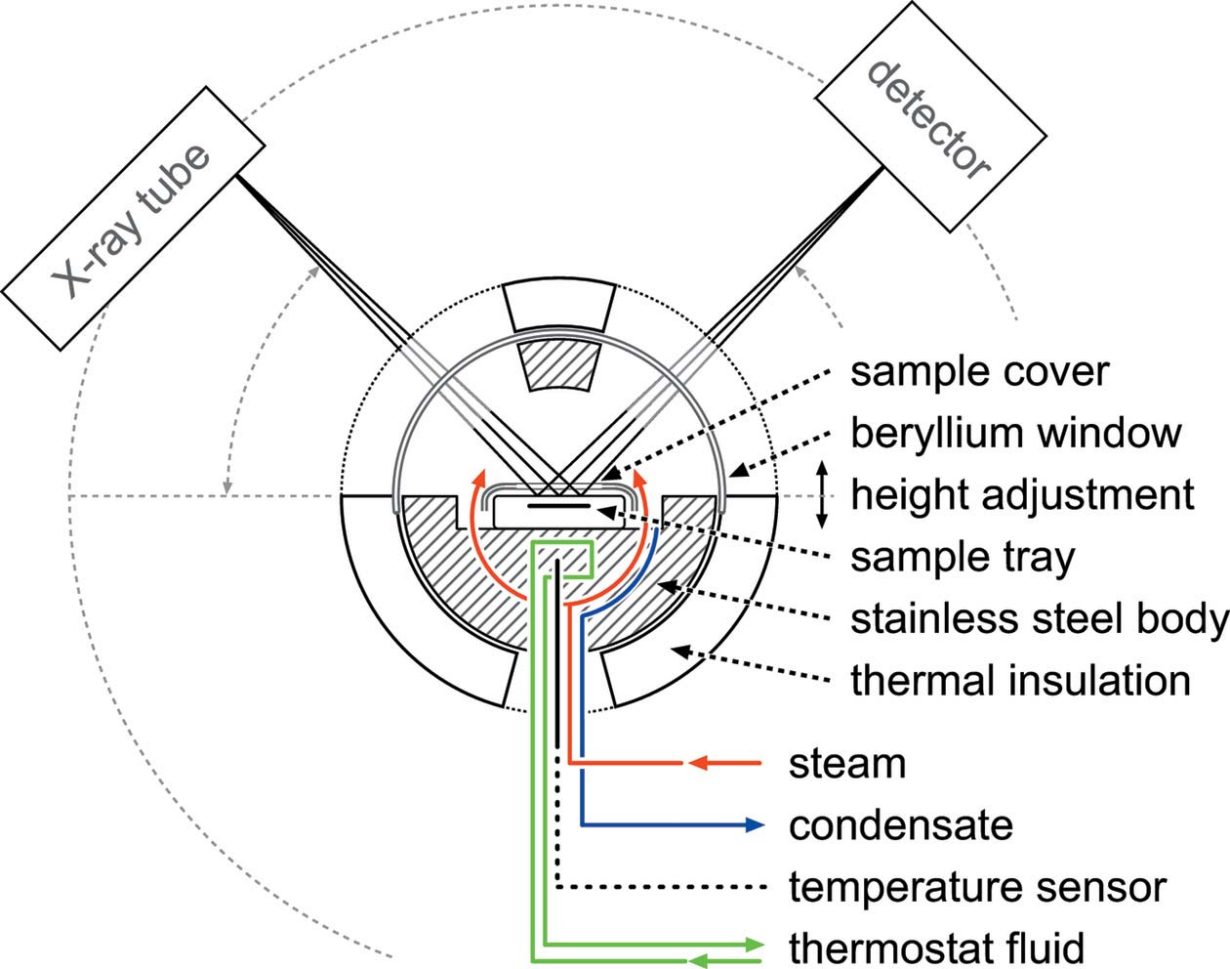
A schematic cross section through the autoclave chamber, a flat-plate chamber for temperatures <483 K and saturated vapour pressure <2 MPa. The front face screw-on lid is not shown in this figure.

The autoclave chamber (Fig. 1 ▸) is a device constructed by Anton Paar for temperatures <483 K and saturated vapour pressure <2 MPa. It is a flat-plate chamber comprising a stainless steel body, thermal insulation and a front face screw-on lid. X-rays are able to pass through beryllium windows protected by Kapton polyimide foil; the associated intensity loss is *ca* 45% under ambient conditions. The formation of condensate droplets on the inside is minimized by applying a surfactant solution prior to each experiment. The chamber is mounted in the middle of the goniometer and can be adjusted in height by ±3 mm to compensate for different specimen thicknesses [Fig. S1(*e*) in the supporting information]. Because of the design the temperature has no significant influence on the Bragg–Brentano geometry. A slight tilt can be corrected by adjusting the θ offset.

Saturated steam is supplied from an external boiler equipped with a tray of calcium hydroxide for scrubbing carbon dioxide. As the steam enters the chamber through the stainless steel body, a homogeneous temperature is maintained, which is measured below the specimen by a PT100 sensor. Constant conditions can be achieved with a precision of ±0.5 K. Pressure is measured at the condensate outlet by an analogue gauge with an accuracy of ±0.025 MPa. Condensate can drain through a vent or reflux to the steam supply boiler supported by an oscillation pump. Separate tubing within the stainless steel body is connected to an external circulation thermostat. The respective pipes are fitted to the diffractometer (in this case a Bruker D8 Advance) without modification of the safety enclosure (here a D8 Discover enclosure).

In the Bragg–Brentano geometry an even specimen surface is crucial for obtaining valid X-ray diffraction (XRD) data. Cracking, buckling or tilting of the surface may obstruct ideal focusing conditions. Therefore cementitious mixtures are favourable to achieve these conditions. A stable surface is generally obtained after 12 h of hydration prior to measurement. The sample trays constructed for the device are 40 × 40 × 14 mm, made out of nickel, and available with specimen depths of either 12 or 2 mm. The 12 mm deep trays can be partially filled with hollow stainless steel cylinders [Fig. S1(*a*)] to reduce the amount of sample needed and to improve surface stability. Condensate leaching is prevented by a curved sample cover made out of 0.5 mm poly(tetrafluorethylene) foil featuring a 40 × 13 mm slot for X-rays.

### 2.2. Sample preparation   

A test sample was prepared analogously to industrial AAC production with a water-to-solid ratio of 0.73 and 48.3% quartz (99.1% SiO_2_; D50 23 µm), 38.1% ordinary Portland cement, 8.7% lime (93% CaO), 5.0% calcium fluorite (>99%; sintered at 973 K for 3 h) and 0.1% aluminium powder. This slurry was poured directly into the sample trays and an even surface obtained by covering the sample trays with thread-seal tape, polyethylene (PE) foil and a planar weight. Analogously to AAC production, the added aluminium powder induces hydrogen gas release, and subsequent foaming yields a thickness of 3–5 mm. The viscosity increases rapidly and the sample is expected to set within 1 h. For further cement hydration, it was kept at 343 K for 7 h and afterwards at room temperature for a total of two days prior to *in situ* measurement. Moisture loss was prevented by the PE foil [Fig. S1(*b*)].

### 2.3. *In situ* measurement   

The Bruker D8 Advance used in this study was equipped with a conventional X-ray source (Cu 40 kV, 40 mA), a position-sensitive detector (LYNXEYE, opening degree 2.56°) and an Ni *K*β filter. A fixed divergence slit of 2 mm on the primary beam yields an irradiated area of *ca* 13 × 11 mm. The sample and its cover were slid into the autoclave chamber [Fig. S1(*d*)], with two cones under the tray matching cavities in the steel body. After sealing of the front face screw-on lid, the chamber was pre-heated (<363 K) using the circulation thermo­stat. With the vent open, steam was flushed through the chamber to ensure saturated water vapour inside the system. Sealing the vent allowed the pressure to build up (0 min). Hydrothermal conditions at 466 K and 1.35 MPa were obtained by steadily increasing the boiler and thermostat temperatures (230 min). After a reaction period of 5.7 h, the steam supply boiler was switched off (570 min) and the system cooled down slowly. *In situ* monitoring was carried out by subsequent θ–2θ measurements from 14.2 to 50.8° 2θ, each taking 13.22 min. A total of 44 out of 51 diffractograms were used for evaluation. Reflection intensities were obtained by exporting areas (c.p.s. × °) for respective 2θ ranges (*DIFFRAC.EVA*; Bruker AXS, Karlsruhe, Germany) and subtracting the background. Normalization was done according to the first or last evaluated measurement. Afterwards, control measurements of the original and then homogenized sample were carried out under ambient conditions.

## 3. Results and discussion   

**Figure 2:**
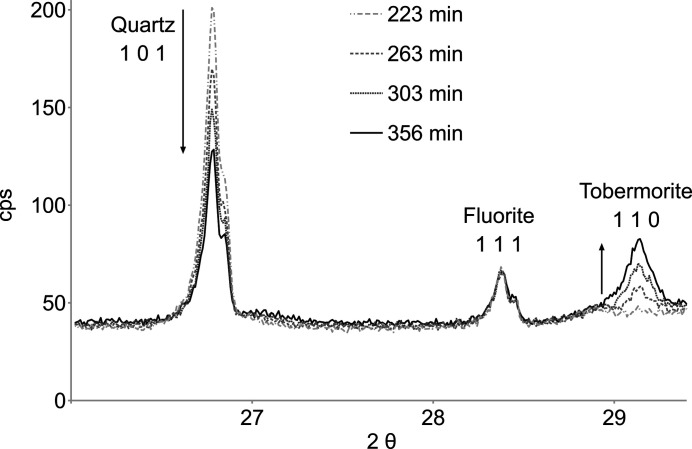
X-ray diffraction results close to and after reaching constant hydrothermal conditions at 466 K and 1.35 MPa (230 min). The tobermorite 110 reflection intensity increases as the quartz 101 reflection intensity decreases. Fluorite serves as internal standard. (See Fig. S2 in the supporting information for the full range.)

Fig. 2 ▸ shows four diffractograms ranging from 26 to 29.3° 2θ, close to and at 466 K (>230 min). Changes in the reflection intensities of quartz 101 [SiO_2_, Powder Diffraction File (PDF; International Centre for Diffraction Data, Newtown Square, Pennsylvania, USA; http://www.icdd.com) card 46-1045; Kern & Eysel, 1993 ▸] and tobermorite 110 [Ca_4.5_Si_6_O_14_(OH)_3_ 2H_2_O, PDF card 83-1520; Hamid, 1981 ▸] indicate that a hydrothermal reaction takes place. Fluorite 111 (CaF_2_, PDF card 35-0816; Swanson *et al.*, 1985 ▸) is seen to be essentially inert and serves as an internal standard.

**Figure 3:**
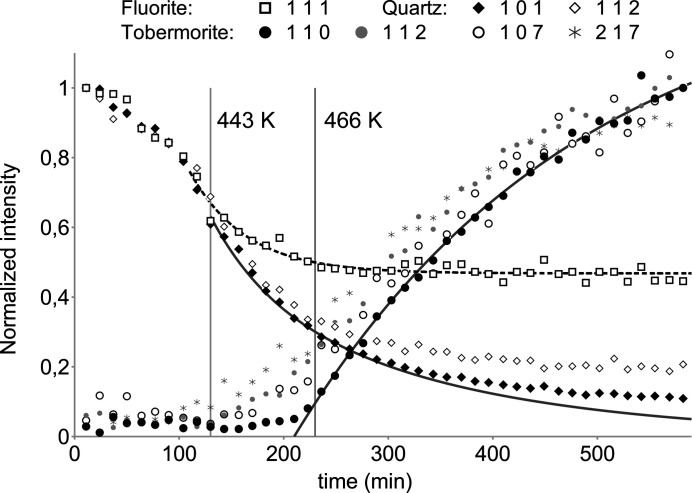
Time-resolved normalized reflection intensities from 373 K (0 min) to constant conditions at 466 K and 1.35 MPa (>230 min). Below 443 K, the fluorite and quartz intensities decrease proportionally in response to increasing temperature and pressure, while above 443 K further decrease of the quartz intensities is related to hydrothermal dissolution. The approximation (solid line) is a combination of temperature correction (dashed line) and a first-order reaction behaviour. Distinct tobermorite crystallization starts at 210 min and a first-order reaction behaviour can be assumed (solid line).

In Fig. 3 ▸ normalized reflection intensities are shown. Below 443 K the fluorite and quartz intensities decrease proportionally in response to increasing temperature and pressure. The steam density inside the autoclave chamber changes from 0.59 kg m^−3^ at 373 K to 6.86 kg m^−3^ at 466 K (Harvey, 1998 ▸), which is accompanied by an increase in X-ray absorption. Up to 466 K (230 min) the fluorite reflection intensity decreases by *ca* 50%. It is fitted by an empirical exponential function (dashed line) and used to make a temperature correction. Above 443 K, the quartz intensity decreases significantly faster than the fluorite intensity, which marks the beginning of hydrothermal dissolution. The quartz dissolution can be approximated as a first-order reaction behaviour after temperature correction (*k* = 7.8 × 10^−5^ s^−1^, solid line). The intensities in the 2θ range of tobermorite reflections start to increase at >443 K (>130 min), with a distinct increase of the 110 reflection starting at 210 min, and then a continuous increase according to a first-order reaction behaviour (*k* = 6.1 × 10^−5^ s^−1^, solid line). In compliance with previous studies, tobermorite forms via poorly crystalline calcium silicate hydrates, initially limited by the dissolution of quartz (Matsui *et al.*, 2011 ▸).

**Figure 4:**
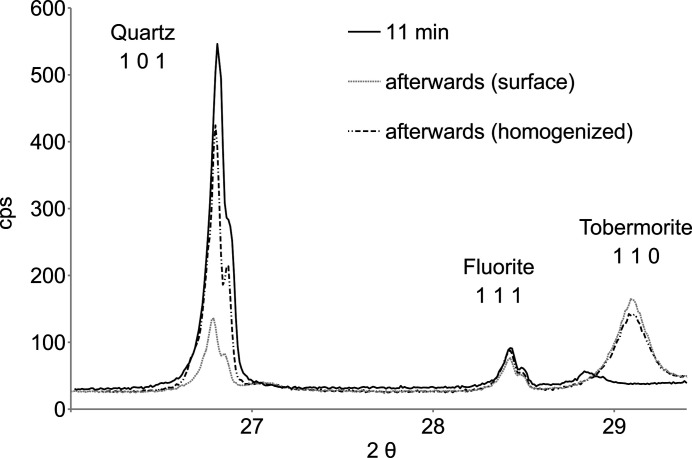
Plots of θ-corrected X-ray diffraction results before (11 min) and after hydrothermal reaction. Homogenization significantly increases the intensity of the quartz 101 reflection, indicating some surface sensitivity of the *in situ* measurements. (See Fig. S3 for the full range)

To interpret these reaction rates, surface sensitivity has to be considered. At an incident angle of 20° the penetration depth is expected to be less than 120 µm. Despite a constant steam density at 466 K (>230 min), Fig. 3 ▸ shows a slight decrease in the fluorite 111 reflection intensity. This may reflect the preferred growth of tobermorite crystals on the sample surface, which could mask fluorite and quartz. Accordingly, in Fig. 4 ▸ the intensity of the fluorite 111 reflection after hydrothermal reaction is lower than before. Upon homogenization, fluorite 111 regains its original reflection intensity while tobermorite 110 remains lower in intensity. Homogenization is accompanied by a notable increase in the intensity of the quartz 101 reflection, which could be related to the initial gravitational settling of larger less-reactive quartz grains. Therefore, the reaction rates derived from the decrease of quartz 101 reflection intensities could be overestimated.

## 4. Conclusions   

This reflection setup is suitable for *in situ* monitoring of hydrothermal reactions and can provide routine information for time-resolved phase quantification. However, in contrast with transmission setups, there are limitations in time resolution, lower signal-to-noise ratios, additional temperature influences and limitations in penetration depth. An internal standard can be used to reveal migration within the sample, correct the influence of temperature on reflection intensities or quantify amorphous content by Rietveld analysis. Due to differences in experimental setup, reaction rates might differ from previous studies of cementitious mixtures in association with the production of autoclaved aerated concrete (Kikuma *et al.*, 2010 ▸; Matsui *et al.*, 2011 ▸). However, the surface sensitivity of this reflection setup could provide additional spatial information on the nature of hydrothermal reactions at interfaces. *In situ* monitoring of other systems is possible if, in accordance with Bragg–Brentano geometry, a stable and even surface can be obtained.

## 5. Supporting data   

Measurement data are available at https://drive.google.com/open?id=1vKxa3fHu92Cc03_U3eGD43c5m2R5EMpK.

## Supplementary Material

Additional figures. DOI: 10.1107/S1600576720006019/gj5245sup1.pdf
