# Supplementary material for: In situ monitoring of hydrothermal reactions by X-ray diffraction with Bragg–Brentano geometry
Source: J Appl Crystallogr. 2020 Jun 18;53(Pt 4):1163–6. doi: 10.1107/S1600576720006019 (PMC7401785; doi:10.1107/S1600576720006019)
Supplement: Supplementary file 1 [file j-53-01163-sup1.pdf]

JOURNAL OF
APPLIED
CRYSTALLOGRAPHY

Volume 53 (2020)

Supporting information for article:

In situ monitoring of hydrothermal reactions by X-ray diffraction with Bragg–Brentano geometry

Karsten Mesecke, Winfried Malorny and Laurence Warr

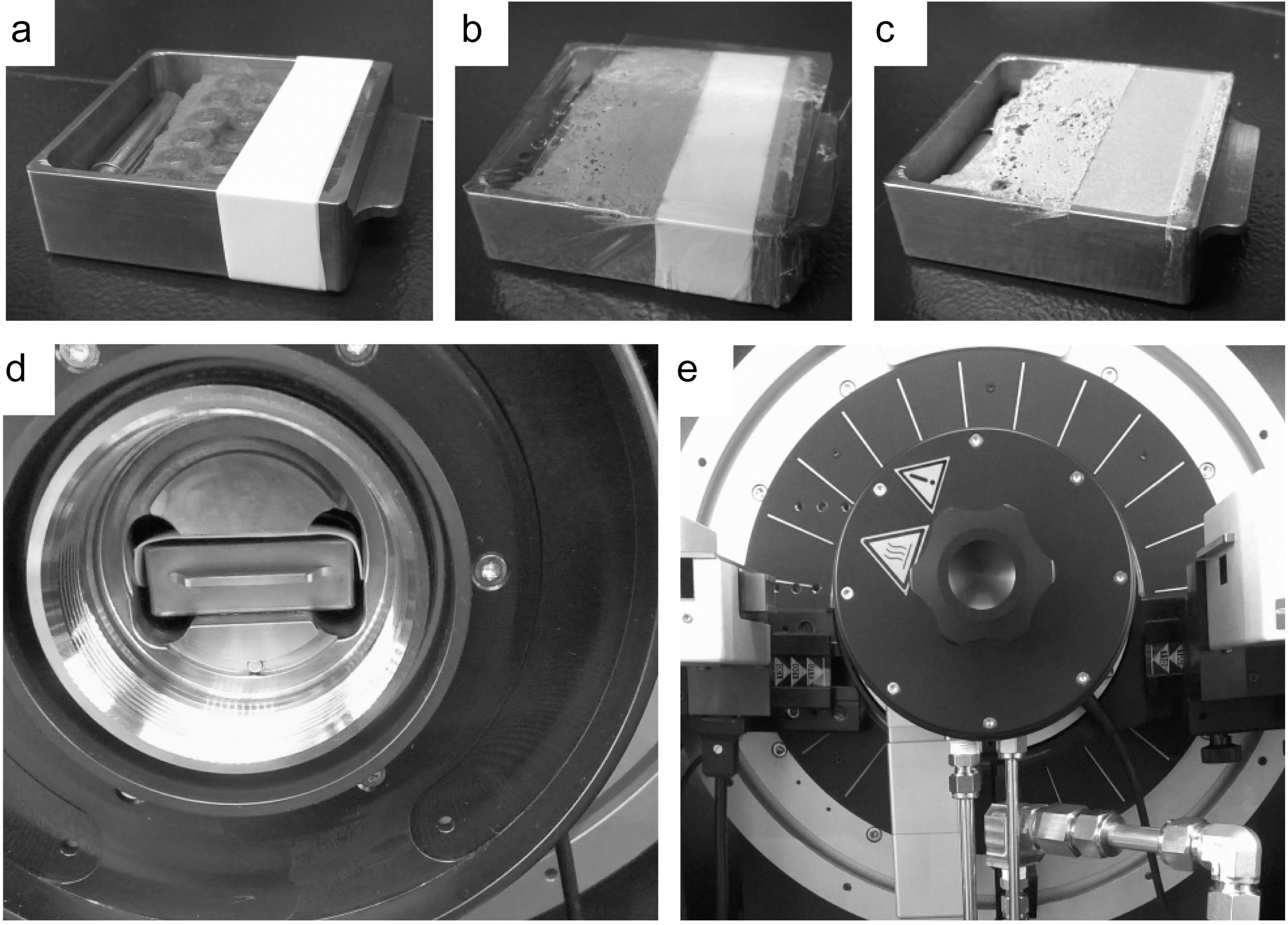

Figure S1. Sample tray with hollow stainless steel cylinders (a) prior casting the slurry, (b) tray wrapped in PE foil during hydration and (c) sample state before loading. Autoclave chamber (d) loaded with a sample and its cover and (e) closed with the screw-on lid.

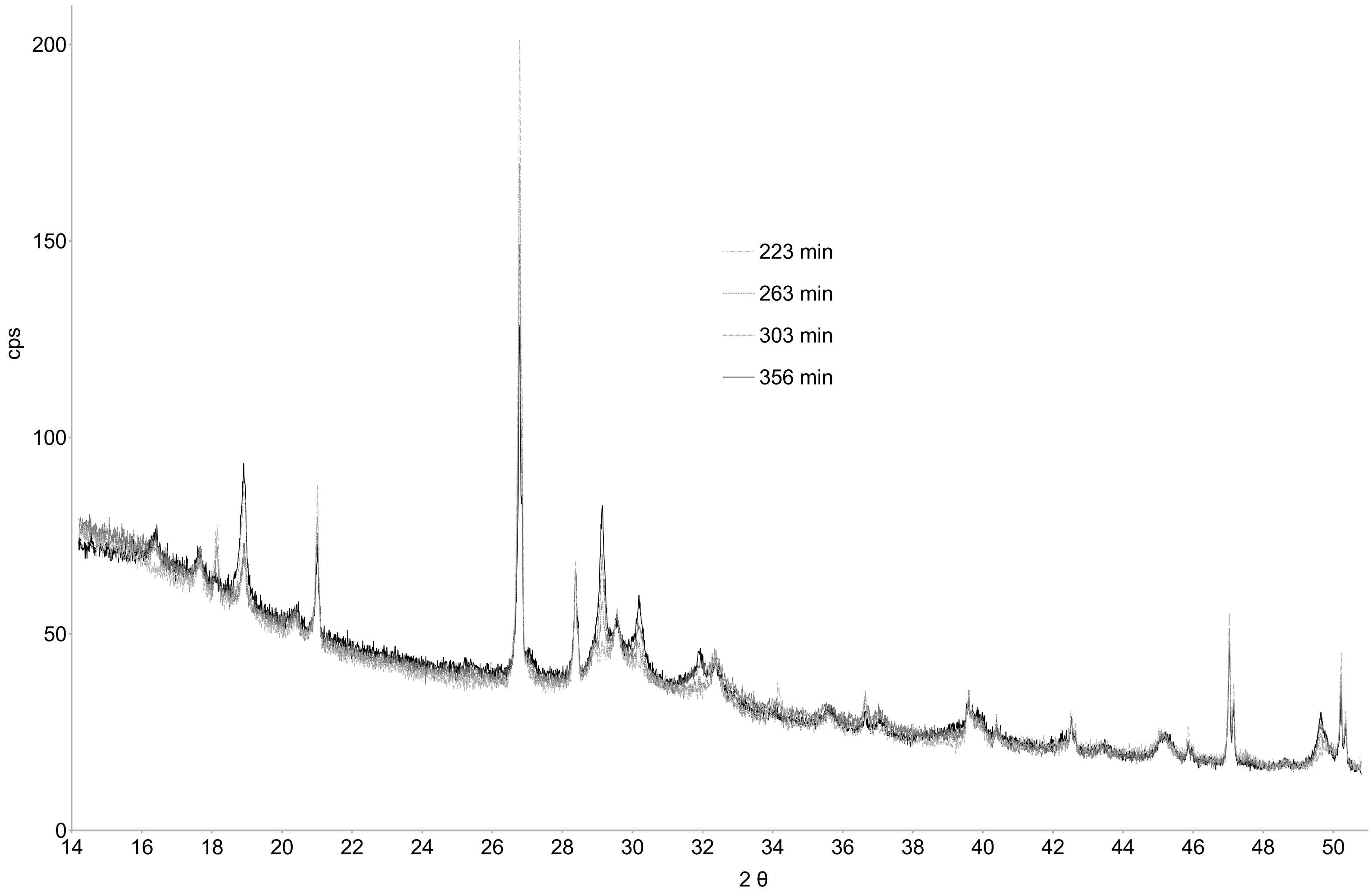

Figure S2. Full range for the plots in Fig. 2.

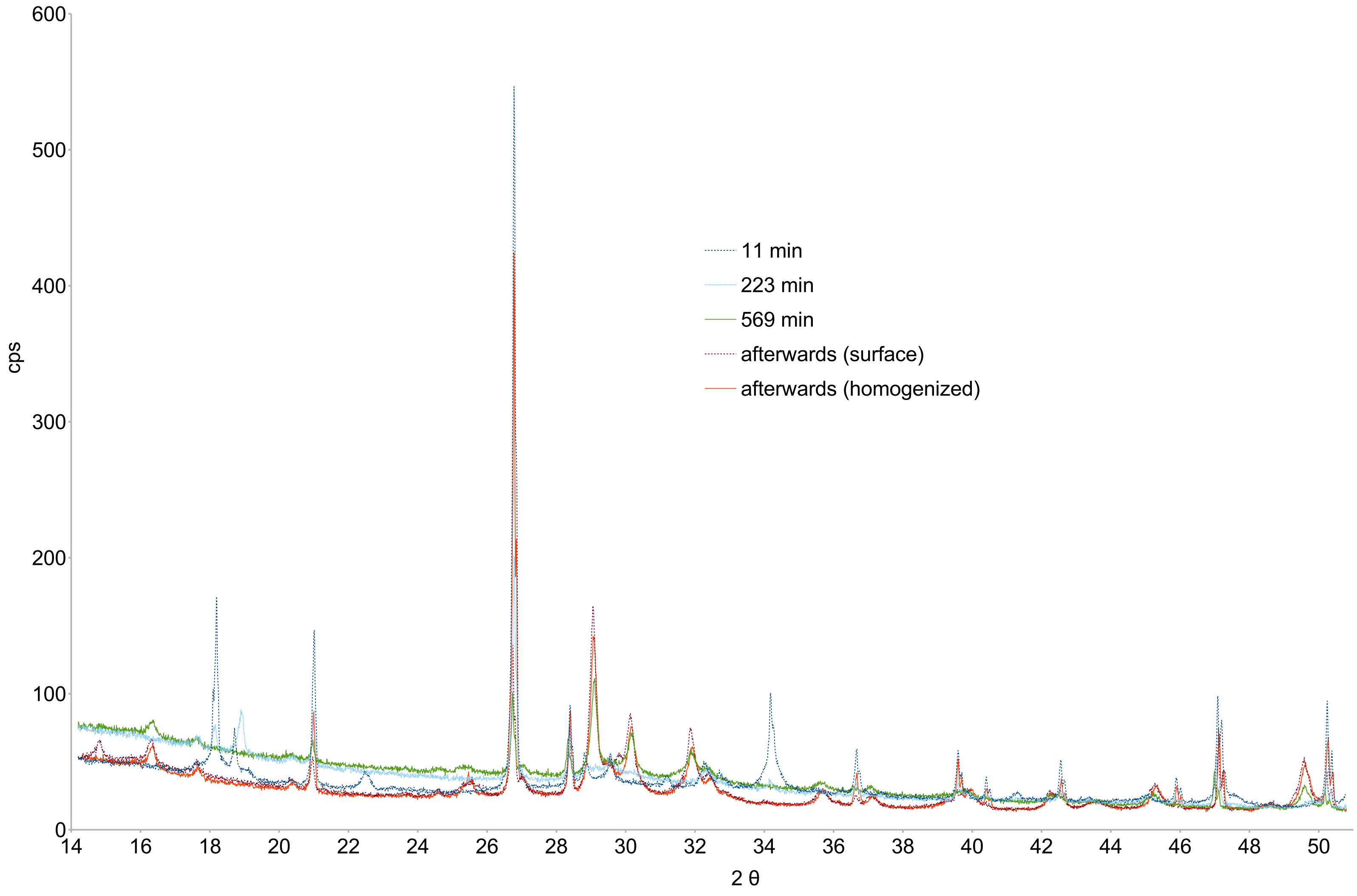

Figure S3. Full range for the plots in Fig. 4.

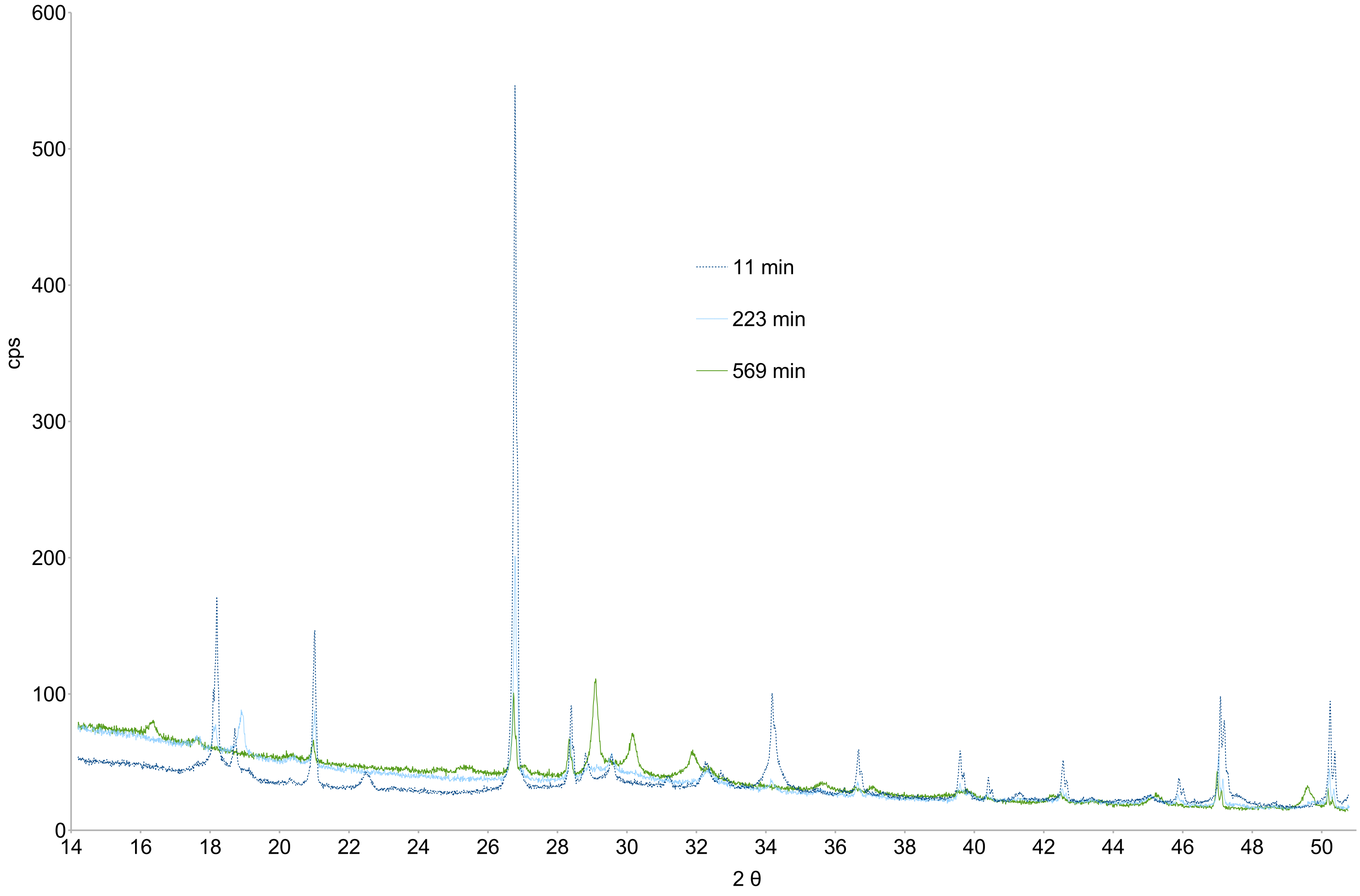

range diffractograms2.eps
IUCr macros version 2.1.11: 2019/01/14

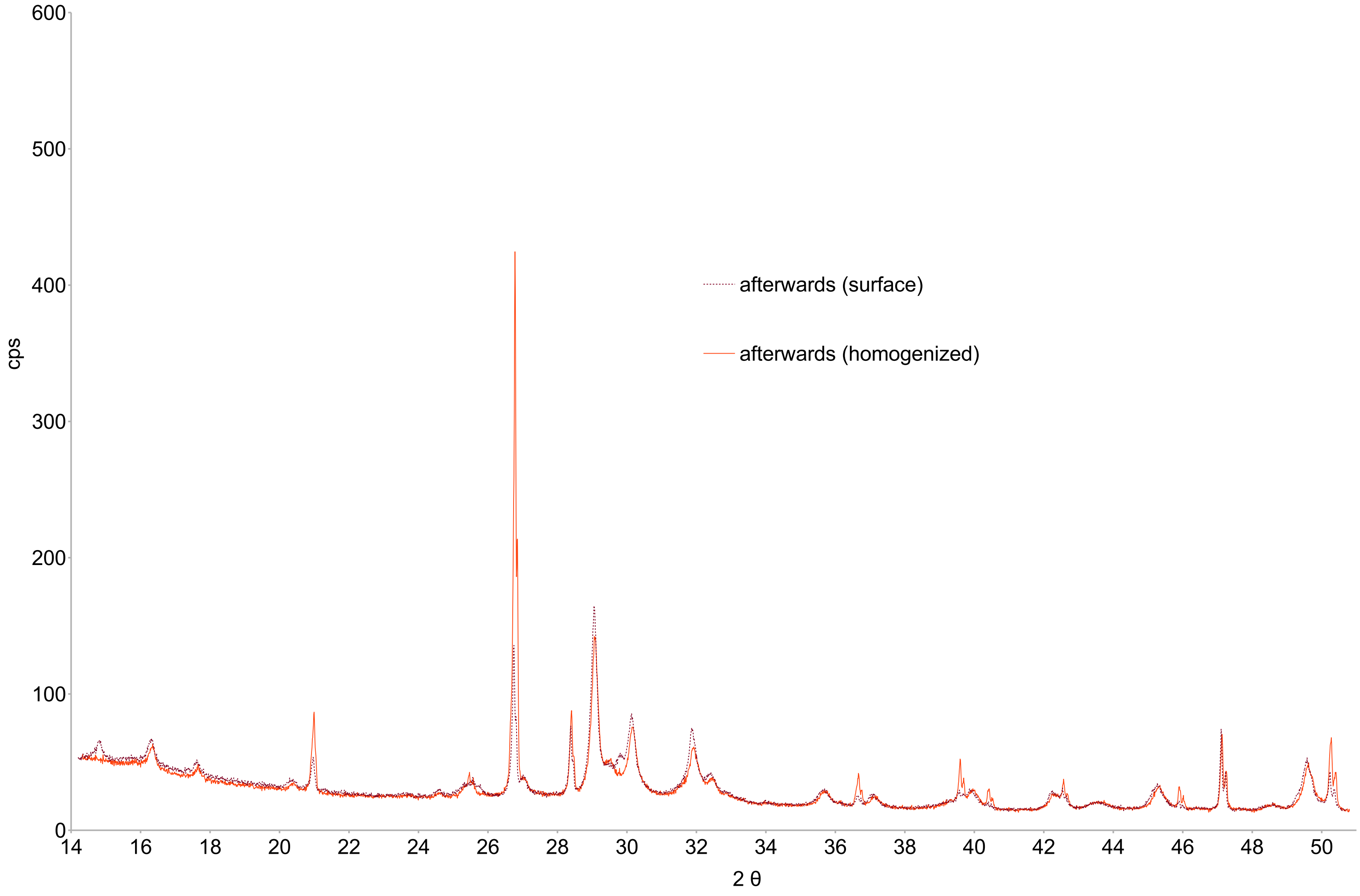

range diffractograms3.eps
IUCr macros version 2.1.11: 2019/01/14

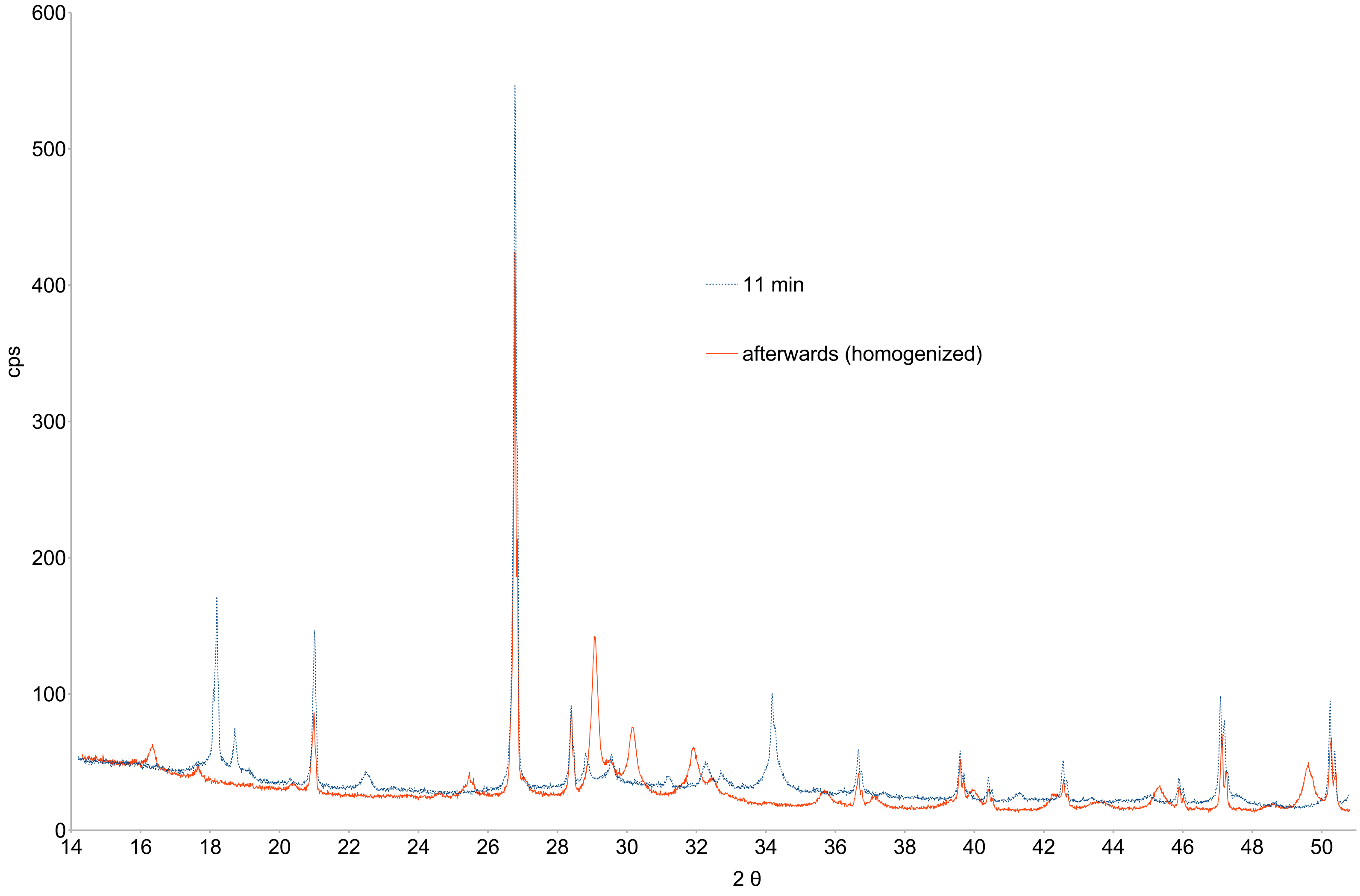

range diffractograms4.eps
IUCr macros version 2.1.11: 2019/01/14
